# Quantitative analysis of dynamic computed tomography angiography for the detection of endoleaks after abdominal aorta aneurysm endovascular repair: A feasibility study

**DOI:** 10.1371/journal.pone.0245134

**Published:** 2021-01-07

**Authors:** Georg Apfaltrer, Francesco Lavra, U. Joseph Schoepf, Marco Scarabello, Ricardo Yamada, Marly van Assen, Akos Varga-Szemes, Brian E. Jacobs, Maximilian J. Bauer, William T. Greenberg, Marcelo Guimaraes, Luca Saba, Carlo N. De Cecco

**Affiliations:** 1 Division of Cardiovascular Imaging, Department of Radiology and Radiological Science, Medical University of South Carolina, Charleston, SC, United States of America; 2 Division of Pediatric Radiology, Department of Radiology, Medical University of Graz, Graz, Austria; 3 Department of Radiology, Azienda Ospedaliero Universitaria (A.O.U.), Cagliari, Italy; 4 Division of Cardiology, Department of Medicine, Medical University of South Carolina, Charleston, SC, United States of America; 5 Postgraduate School in Radiodiagnostics, Universita degli Studi di Milano, Milan, Italy; 6 Division of Vascular Interventional Radiology, Department of Radiology and Radiological Science, Medical University of South Carolina, Charleston, SC, United States of America; 7 Center for Medical Imaging, University Medical Center Groningen, North East Netherlands, Groningen, The Netherlands; Universita degli Studi di Padova, ITALY

## Abstract

**Objectives:**

To assess the feasibility of quantitative analysis of dynamic computed tomography angiography (dCTA) for the detection of endoleaks in patients who underwent endovascular repair of abdominal aortic aneurysms (EVAR).

**Material and methods:**

Twenty patients scheduled for contrast-enhanced CT angiography (CTA) of the abdominal aorta post-EVAR were prospectively enrolled. All patients received a standard triphasic CTA protocol, followed by an additional dCTA. The dCTA acquisition enabled reconstruction of color-coded maps depicting blood perfusion and a dCTA dataset of the aneurysm sac. Observers assessed the dCTA and dynamic CT perfusion (dCTP) images for the detection of endoleaks, establishing diagnostic confidence based on a modified 5-point Likert scale. An index was calculated for the ratio between the endoleak and aneurysm sac using blood flow for dCTP and Hounsfield units (HU) for dCTA. The Wilcoxon test compared the endoleak index and the diagnostic confidence of the observers.

**Results:**

In total, 19 patients (18 males, median age 74 years [70.5–75.7]) were included for analysis. Nine endoleaks were detected in 7 patients using triphasic CTA as the reference standard. There was complete agreement for endoleak detection between the two techniques on a per-patient basis. Both dCTA and dCTP identified an additional endoleak in one patient. The diagnostic confidence using dCTP for detection of endoleaks was not significantly superior to dCTA (5.0 [5–5] vs. 4.5 [4–5], respectively; p = 0.11); however, dCTP demonstrated superior diagnostic confidence for endoleak exclusion compared to dCTA (1.0 [1–1] vs 1.5 [1.5–1.5], respectively; p <0.01). Moreover, the dCTP endoleak index was significantly higher than the dCTA index (18.5 [10.8–20.5] vs. 3.5 [5–2.7], respectively; p = 0.02).

**Conclusions:**

Quantitative analysis of dCTP imaging can aid in the detection of endoleaks and demonstrates a higher endoleak detection rate than triphasic CTA, as well as a strong correlation with visual assessment of dCTA images.

## Introduction

The most common treatment for patients with infrarenal aortic aneurysms is endovascular repair of abdominal aortic aneurysms (EVAR). However, endograft-related complications necessitate repeated interventions in 6% of patients after 1 year, 16% after 3 years, and up to 30% of cases within 6 years after initial surgery [1–5]. Consequently, a long-term post-EVAR follow-up is required in order to rule out the presence of an endoleak, prosthesis migration, or other complications. Of these, type II endoleaks are the most common and types I and III endoleaks are often associated with post-EVAR aneurysm rupture [6–8]. The standard of care imaging modality for the evaluation of pre- and post-EVAR is computed tomography angiography (CTA) [7,9–12]. Recently, advanced dual-source CT systems with alternating table positions (‘shuttle mode’) allow for dynamic acquisition of blood perfusion in the endograft, aneurysm sac, and adjacent aortic branches. Owing to a high temporal sampling rate, this approach enables acquisition of dynamic CT angiography (dCTA) data.

Previous studies have explored the feasibility and diagnostic performance of visually assessing dCTA images in post-EVAR patients. For example, Sommer at al. demonstrated the feasibility of a dCTA protocol to differentiate types of endoleaks [13] and Lehmkuhl et al. showed that dCTA has a higher detection rate for endoleaks compared to the static triphasic CTA [14].

However, a dCTA dataset can also provide color-coded maps and tissue attenuation curves (TACs) to quantify blood perfusion, enabling both the qualitative assessment of the color-coded maps and the quantification of the blood flow (BF) inside the analyzed tissue. To our knowledge, the quantitative information comprised in these data has not been investigated for its value in endoleak detection.

Thus, the purpose of this study was to assess the feasibility of quantitative dCTA analysis for the detection of endoleaks in patients who underwent EVAR.

## Materials and methods

### Patient population

The Institutional Review Board of the Medical University of South Carolina approved this prospective study and written informed consent was obtained from all patients (Protocol# PRO00044160). Between September 2016 and December 2017, a total of 42 consecutive patients scheduled for contrast-enhanced CTA of the abdominal aorta following EVAR were eligible for the study. Patients were included if they were 18–90 years old and were able to comply with all clinical and study procedures. Patients with a glomerular filtration rate < 45 ml/min within 30 days prior to the examination (n = 6) and known adverse reactions to iodine-based contrast material (n = 1) were excluded. Obesity was not considered an exclusion criterion. Fifteen patients declined participation in the study.

A total of 20 patients were eventually included in the study; each underwent a standard tri-phasic CT protocol followed by a dCTA acquisition. Demographic and clinical information were retrieved from medical records.

### CT image acquisition

All examinations were performed using a third generation dual-source CT scanner (Somatom Force, Siemens Healthineers, Forchheim, Germany).

#### Triphasic CTA protocol

The triphasic CT angiography protocol contained a non-enhanced phase acquired in single energy, followed by an arterial and a delayed phase that were both acquired in dual energy mode. This protocol was specifically chosen to maximize the sensitivity of this technique in the detection of all endoleaks. The three static acquisitions were obtained in the cranio-caudal direction at inspiratory breath-hold with a field of view (FOV) extending from the diaphragm to the origin of femoral arteries. The non-enhanced acquisition had the following image parameters: detector configuration, 192 x 0.6 mm; tube voltage, 100 kV (range 90–110); median tube current, 190 mAs [170–201.5]; and pitch, 0.6. The arterial and delayed phases had the following image parameters: detector configuration, 128 x 0.6 mm; tube A voltage, 90 kV; median tube current, 100 mAs [91.5–111]; tube B voltage, 150 kV; median tube current, 59 mAs [55.5–62.5]; and pitch, 0.7. Automated real-time attenuation-based tube current modulation (CARE Dose 4D, Siemens) was used per default to minimize radiation exposure.

The arterial phase scan utilized a bolus tracking technique; the acquisition started seven seconds after a threshold of 100 Hounsfield units (HU) was reached within a region of interest (ROI) in the abdominal aorta at the level of the celiac artery. A total of 50 ml of a low-osmolar, non-ionic iodinated contrast medium (Iohexol, Omnipaque 350 mgI/ml; GE Healthcare, Princeton, United States) was injected through a 20-gauge needle placed in an antecubital vein at a flow rate of 4 ml/s. An 80 ml bolus of saline solution was administered at the same rate after each contrast injection. The delayed phase acquisition started with a standard delay of 70 seconds after the start of contrast material injection.

The triphasic dataset was reconstructed using a soft tissue convolution kernel (Bv36) and an iterative reconstruction technique (ADMIRE, Siemens; strength level 3) with a section thickness of 1.5 mm. A weighting factor of 0.7 was used to reconstruct the arterial and delayed phases in standard linearly blended M_0.7 series, combining 70% of the low with 30% of the high-kV spectrum.

#### Dynamic CTA protocol

A customized shuttle mode was used to obtain the dCTA acquisitions with the same FOV as the triphasic protocol. This modality allows the table to shuttle back and forth between the diaphragm and the origin of femoral arteries; a fast spiral acquisition is performed at short time intervals between these two positions to obtain images across the entire FOV.

The protocol consisted of intermittent image acquisitions performed in the cranio-caudal direction at the following parameters: detector configuration, 48 x 1.2 mm; tube voltage, 70 kV in 18 patients and 90 kV in one patient; tube current, 200 mAs; pitch, 1; and rotation time, 0.32 seconds. This protocol was designed to limit the radiation dose to a maximum dose length product (DLP) of 1065 mGy/cm, and to maximize iodine attenuation and temporal coverage.

A total of 50 ml of Iohexol (Omnipaque 350 mgI/ml; GE Healthcare, Princeton, United States) were administered through a 20-gauge needle placed in an antecubital vein with a flow rate of 4 ml/s, followed by 50 ml of saline solution at the same rate. Two seconds after initiating injection of contrast material, 12 acquisitions were executed (1 scan every 4 seconds), followed by three additional acquisitions (1 scan every 10 seconds). This longer interphase delay favors the visualization of a slow filling pattern, typical of type II endoleaks.

The dynamic dataset was reconstructed using a soft tissue convolution kernel (Bv36) and an iterative reconstruction technique (ADMIRE, Siemens; strength level 3) with a section thickness and increment of 5 mm.

### Image analysis

#### Triphasic CT angiography

A radiologist with 12 years of experience in cardiovascular imaging (C.N.D.C.) interpreted the standard triphasic CT angiography images. The reviewer assessed all image series using multiplanar reformations in a separate workstation (Syngo.via VB10B, Siemens) without knowledge of previous CT reports or clinical data. An endoleak was defined as a hyper-attenuating area inside the aneurysm sac, but external to the graft, that is absent on non-enhanced images. Endoleaks were classified as the following: type I, blood leak at the graft extremities; type II, retrograde blood flow via aortic branch vessels; type III, blood leak caused by structural failure of the stent-graft; and type IV, leak resulting from a porous stent-graft [7].

An objective image quality assessment was performed. Standardized ROI (1 cm^2^) were positioned in homogeneous portions of the aortic lumen, the endoleak and aneurysm sac to quantify attenuation values and contrast to noise ratios (CNRs). For endoleaks < 1 cm^2^, a ROI was freely drawn around the entire endoleak. The standard deviation of the HU values of the unaffected aneurysm sac was regarded as the image noise (IN). The following formulas were used to estimate the CNRs of the aortic lumen and the endoleak [13]:

CNR_AL_ = HU_AL_−HU_AS_ / IN_AS_

CNR_EN_ = HU_EN_−HU_AS_ / IN_AS_

HU_AL_ represents the HU values of the aortic lumen. HU_AS_ and IN_AS_ are the HU and the image noise of the aneurysm sac, respectively. HU_EN_ is the HU of the endoleak.

Subjective image quality was also completed according to the following 5-point Likert scales: contrast enhancement (from 1 = poor to 5 = excellent), image noise (from 1 = high level of image noise to 5 = low level of image noise), and suitability for the detection of endoleaks (from 1 = non-diagnostic scan to 5 = excellent for the assessment of endoleaks) [15].

#### Dynamic CT angiography

The dynamic dataset was analyzed using a dedicated tool (CT Dynamic Angio, Syngo.via VB10B, Siemens) on a standard workstation, while a motion correction tool was applied to align corresponding sections in different phases. The dCTA images were displayed in cine mode with dynamic presentation of the axial sections, focusing on frames in which every slice position was on the z-axis. Randomization software was used to select the patients’ images for analysis. All acquisition phases were analyzed twice by two observers (M.S/F.L) with 4 and 5 years of experience in cardiovascular imaging, respectively, with >1 month between each evaluation. The observers were blinded to clinical data and previous CT reports. The observers were also blinded to the results of the first evaluation at the time of the second analysis. Endoleaks were assessed according to the aforementioned criteria [7]. The time spent on overall analysis of the dCTA images was recorded. The time at peak of contrast enhancement of the aortic lumen and the endoleak was recorded, as well as the difference of these times.

In addition, an endoleak index was calculated to quantify the capability of this technique to discriminate the endoleak from the surrounding thrombotic tissue:

Dynamic Endoleak index = HU_EN_ / HU_AS_

The two observers also performed an objective image quality assessment as explained above, applying the measurement to the phase with greatest enhancement in the aortic lumen [13]. The aforementioned 5-point Likert scales were used to assess subjective image quality [15]. To avoid recall bias, the two observers quantified their diagnostic confidence in the identification of endoleaks during their first interpretation using the following Likert scale: 1 = certain absence, 2 = probable absence, 3 = possible presence, 4 = probable presence, and 5 = certain presence [15].

#### Dynamic CT perfusion

**Fig 1:**
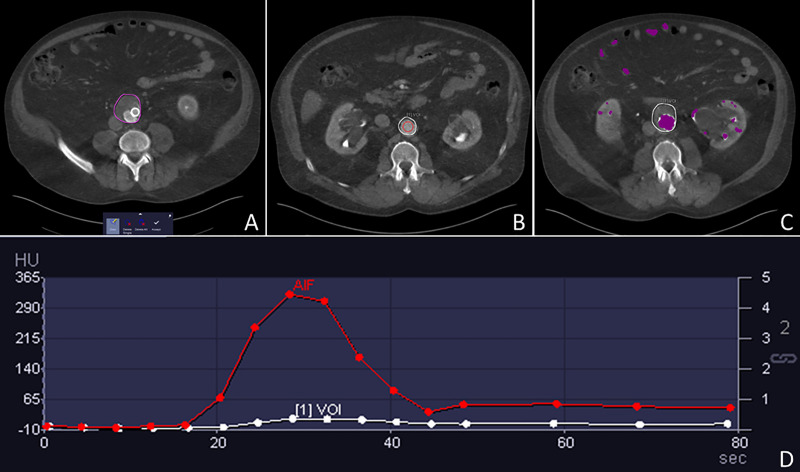
Post-processing steps involved using the CT Myocardial Perfusion software. 70-year-old man status post EVAR 3 years prior to the dCTA examination. After an initial manual segmentation step of the aorta (A), segmentation propagates automatically. A ROI is positioned in the aortic lumen (B) which is then automatically defined (C) in order to compute the AIF (D).

The CT Myocardial Perfusion software (Singo.via VB10B, Siemens) was used to process the dCTA datasets with application of motion correction and 4D noise reduction. A volume of interest was manually defined to encompass the portion of the abdominal aorta containing the endo-prosthesis and the aneurysm sac. This volume was then automatically segmented. A ROI was placed in the abdominal aorta in a cranial position with respect to the stent-graft to define an arterial input function (AIF), i.e. the concentration of contrast medium in the supplying artery. Subsequently, the aortic lumen was automatically defined according to HU threshold values set by the software (Fig 1).

The software exploits a dedicated parametric deconvolution technique that uses the intravascular and extravascular space to generate TACs (i.e. the concentration of contrast media in the thrombotic tissue over time). Therefore, the algorithm defines the upslope from the TAC for every voxel and computes the blood flow (BF) as stated by the following equation [16]:

BF = _Max_Upslope (TAC)/ Maximum (AIF)

Using this quantitative information, a color-coded map of BF was automatically created. The same two observers qualitatively analyzed these color maps twice according to the same diagnostic criteria mentioned above. A ROI was placed at the level of suspected endoleak and analysis of the corresponding TAC and AIF curve confirmed diagnosis. The BF values of the endoleak and the total time spent on each evaluation (e.g. reconstruction of the perfusion maps) were annotated. Moreover, the software automatically calculated the time at peak enhancement of the aorta and the endoleak, and the difference of these was recorded. Another ROI (1cm^2^) was positioned in a homogeneous portion of the aneurysm sac and the corresponding BF value was recorded. Next, a CT perfusion endoleak index was calculated using the following formula:

BF endoleak index = BF_EN_ / BF_AS_

Where BF_EN_ and BF_AS_ represent the blood flow in the endoleak and in the aneurysm sac, respectively.

The image noise and contrast were evaluated for the overall subjective image quality assessment using the following Likert scale: 1 = very poor, 2 = scarce, 3 = reduced, 4 = good, and 5 = excellent.

Diagnostic confidence for determining the presence of an endoleak was rated in the first interpretation session as mentioned above.

### Dose length product and effective radiation dose

DLP of each scan was recorded, and effective radiation dose was estimated based on a conversion factor of 0.015 mSv · mGy^-1^ · cm^-1^ [17].

### Statistical analysis

All analyses were performed with SPSS 24 (SPSS Inc., Chicago, IL, USA). Normally distributed data are reported as mean ± standard deviation and not normally distributed variables as median with interquartile range (IQR) in square brackets [Q_1_ –Q_3_]. Data was tested for normality using the Shapiro-Wilk test. The student t-test was used to compare normally distributed variables. The Mann-Whitney U test was used to compare age, gender and body mass index (BMI) between patients. With respect to the diagnostic confidence, scores of 1 and 2 indicated absence of an endoleak and scores of 3–5 implied presence of an endoleak [15]. The Wilcoxon test was used to compare the following: endoleak index, the time to each peak and their difference, the diagnostic confidence for presence and absence of an endoleak between dCTA and dynamic CT perfusion (dCTP), and the radiation dose between the tri-phasic CTA and the dynamic acquisition. The inter- and intra-observer agreement for the detection of endoleaks by dCTA and dCTP were evaluated using linearly weighted Kappa (κ) statistics. The results were interpreted as follows: poor, κ<0.20; fair, κ = 0.21–0.40; moderate, κ = 0.41–0.60; good, κ = 0.61–0.80; and excellent, κ>0.80. A p-value < 0.05 was considered significant for all tests.

## Results

### Patient population

Among the 20 patients included in the study, one was excluded because of an incomplete dynamic examination due to contrast media extravasation. A total of 19 patients (18 males, median age of 74 [70.5–75.7], mean BMI 29 ± 4.7 kg/m^2^) successfully concluded all clinical and study procedures. There was no statistical difference between patients with and without endoleaks in terms of age, gender and body mass index (all p > 0.05). The median follow-up time was 542 [IQR: 42–1241] days after EVAR. Six of the CT follow ups occurred within 1 year (32 [IQR: 29–78] days) and 13 patients had follow ups after 1 year (875 [IQR: 542–1260] days). Due to an associated enlargement of the aneurysm sac, four patients underwent selective catheter angiography with endoleak embolization during the follow-up period and prior to enrollment in the study. Table 1 shows the different types of endografts used. Two different types of endografts were simultaneously implanted in three patients.

**Table 1:** Types and dimensions of the implanted endografts.

Endoprosthesis	n	Length (mm)	Diameter (mm)
Endurant (Medtronic)	6	148.7 ± 22	17.7 ± 5.7
Endurance II Bif (Medtronic)	2	116.7 ± 11.8	17.7 ± 5.7
Excluder (Gore)	5	119.8 ± 42	22.8 ± 6.4
Zenith (Cook)	3	98.3 ± 30.3	25.2 ± 10.4
Zenith Spiral (Cook)	1	73	23
Zenith Flex (Cook)	2	92	30
Gore Viabahn (Gore)	2	109	32

Data are expressed as means ± standard deviations.

### Subjective and objective image quality

The results of the objective and subjective quality assessment for triphasic and dCTA are shown in Table 2. The CNR of the aortic lumen for dCTA was significantly superior to that of the arterial phase of standard CTA (p <0.001). Conversely, the CNR of endoleaks were not significantly different between the two techniques (p = 0.14). The overall subjective image quality of dCTP images was very good, with a median value of 4.7 [4.5–5].

**Table 2:** Image quality assessment of tri-phasic and dynamic CTA images.

	CNR		Subjective image quality		
	Aortic lumen	Endoleak	Contrast	Noise	Diagnostic
Arterial Phase Tri-phasic CTA	15.3 ± 5.4	5.4 ± 1.8	4.8 ± 0.2	4.1 ± 0.3	4.6 ± 0.3
Delayed Phase Tri-phasic CTA	3.3 ± 1	2.8 ± 1	3.7 ± 0.2	3.7 ± 0.3	3.9 ± 0.3
Dynamic CTA	24.7 ± 12	5 ± 3.3	4.7 ± 0.4	3.8 ± 0.6	4 ± 0.4

Values are expressed as mean ± standard deviations.

### Endoleak detection rate and endoleak index for dynamic CTA and CTP

A total of seven patients were found positive for endoleak with standard CTA, all of which were type II. The triphasic CTA detected a total of nine endoleaks in those 7 patients, while dCTA and dCTP discovered 10 and 11, respectively. Table 3 shows the distribution of findings for the evaluation of dCTA and dCTP in relation to standard CTA.

**Table 3:** Summary of endoleaks identified with dynamic CTA and CTP in relation to the standard CTA.

Patient number	Endoleaks by Dynamic CTA (n)				Endoleaks by Dynamic CTP (n)				Standard CTA
	A1	A2	B1	B2	A1	A2	B1	B2	
1	0	0	0	0	0	0	0	0	0
2	2	2	2	2	2	2	2	2	2
3	2	2	2	2	2	2	2	2	1
4	0	0	0	0	0	0	0	0	0
5	2	2	2	2	3	3	2	3	2
6	0	0	0	0	0	0	0	0	0
7	1	1	1	1	1	1	1	1	1
8	1	1	1	1	1	1	1	1	1
9	1	1	1	1	1	1	1	1	1
10	0	0	0	0	0	0	0	0	0
11	0	0	0	0	0	0	0	0	0
12	0	0	0	0	0	0	0	0	0
13	0	0	0	0	0	0	0	0	0
14	0	0	0	0	0	0	0	0	0
15	0	0	0	0	0	0	0	0	0
16	0	0	0	0	0	0	0	0	0
17	0	0	0	0	0	0	0	0	0
18	0	0	0	0	0	0	0	0	0
19	1	1	1	1	1	1	1	1	1
Totals	10	10	10	10	11	11	10	11	9

A1 and B1, first reading, A2 and B2 second reading or observer A and B, respectively.

On a per-patient basis, there was complete agreement between triphasic CTA, dCTA and dCTP in the detection of endoleaks. The median time (in seconds) at the peak of the aortic lumen and endoleak, as well as the difference in these times, were 24.3 [0.4–28.2], 36.2 [32.6–42.9] and 8.9 [7.9–17.3] for dCTA, and 24.3 [20.5–28.4], 37.5 [33.6–40] and 10.3 [8.2–15.1] for dCTP, respectively. There was no significant difference in these values between techniques (p = 0.18, p = 0.14 and p = 0.14, respectively).

**Fig 2:**
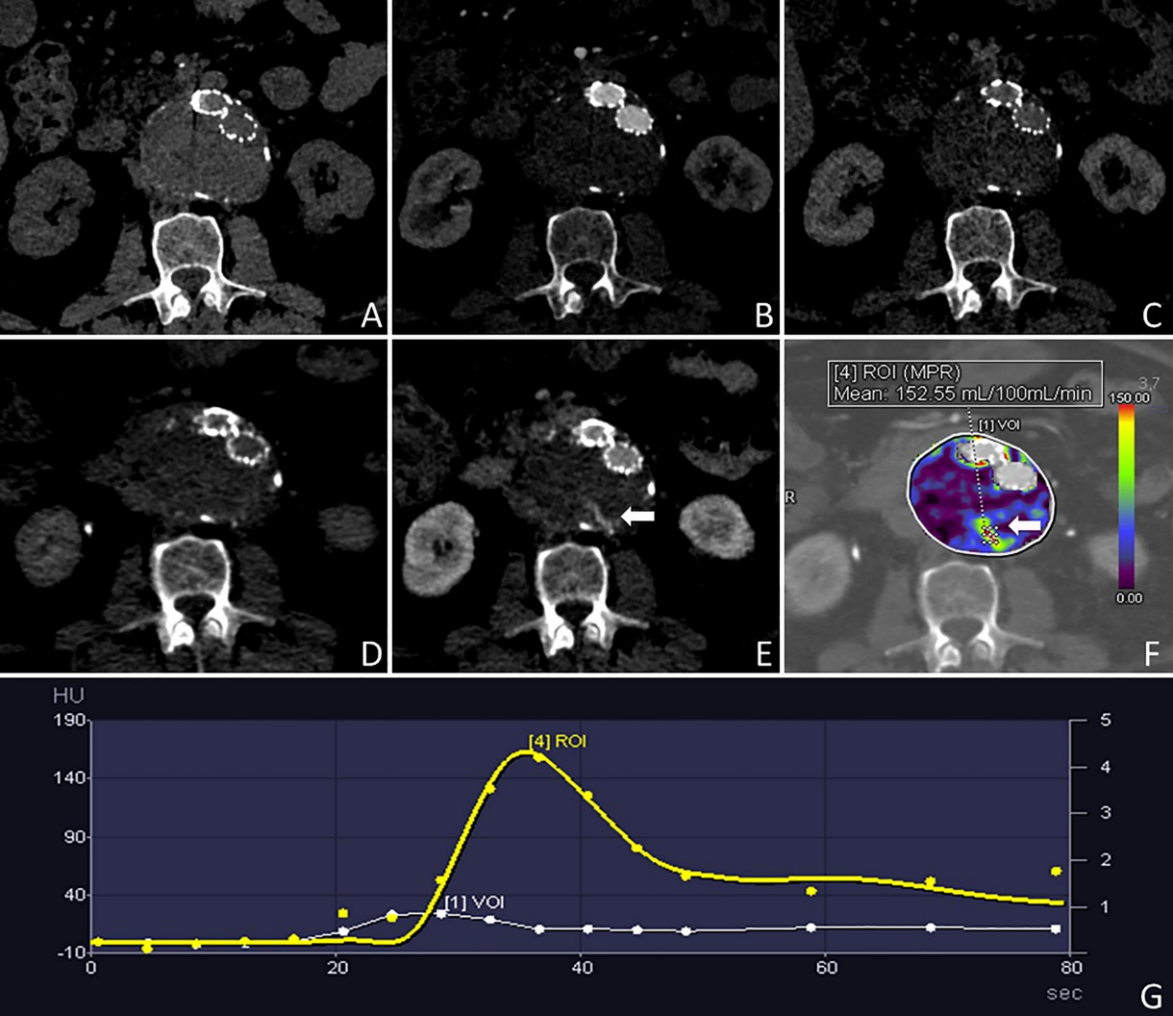
Case demonstration. 76-year-old man with a BMI of 30.85 kg/m^2^ who had undergone EVAR 2.5 years prior to the CTA examination. The non-contrast, arterial and venous static CTA acquisitions (A, B and C, respectively) do not show the presence of any endoleaks. The dCTA images acquired 2 and 38 seconds after the contrast media injection (D and E, respectively) show a type II endoleak, fed by a lumbar artery, that is also depicted by the color-coded perfusion maps (arrow in E and F). The TAC shows the kinetics of the endoleak (G) characterized by a wash-in that starts after 20 seconds, reaching a peak at 36 seconds, and then a rapid wash-out that does not allow its detection with the static CTA acquisitions.

**Fig 3:**
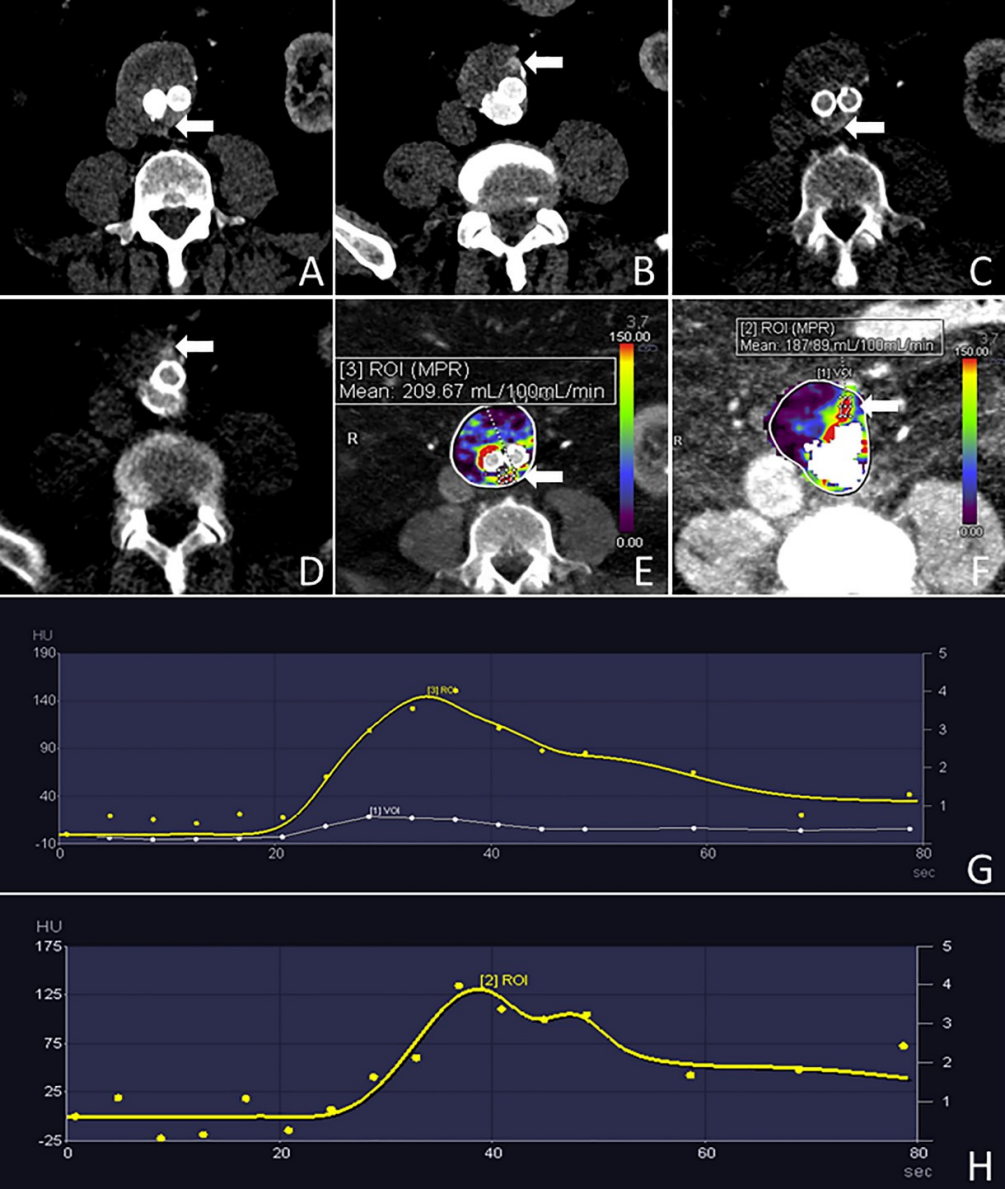
Case demonstration. 74-year-old man with a BMI of 33.51 kg/m^2^ who had undergone EVAR 1.5 years prior to the CTA examination. The arterial phase of the static CTA shows a type II endoleak fed by a lumbar artery and the inferior mesenteric artery (arrows in A and B, respectively). This is also depicted by the dCTA study during the acquisition performed at 38 seconds after contrast media injection (arrows in C and D). Panels E and F show the color-coded perfusion maps and the TACs (G and H). The TAC (G) corresponding to the cranial portion of the endoleak fed by a lumbar artery (arrow in E) shows a kinetic characterized by a rapid wash-in, a time to peak at 36 seconds, and a delayed washout. The TAC (H) corresponding to the caudal portion of the endoleak fed by the inferior mesenteric artery (arrow in F) shows a kinetic characterized by a rapid wash-in, two time to peaks at 38 and 48 seconds, and a delayed washout.

The discrepancy between the number of endoleaks identified by standard CTA and those detected by the dCTA and dCTP was caused by different results provided by the three techniques in two patients. For one patient in particular, standard CTA identified only one endoleak, while both the dCTA and dCTP were able to identify an additional endoleak characterized by a time to peak of 36 seconds and a rapid washout (Fig 2). Moreover, triphasic CTA and dCTA identified two endoleaks in another patient; however, the observers classified one of these to be composed of two different lesions during 3 out of 4 reading sessions of dCTP images. This type II endoleak was fed by a lumbar artery in its cranial portion and by the inferior mesenteric artery in its distal end, presenting two time peaks at 38 and 48 seconds, respectively (Fig 3).

The median HU values in the endoleak and aneurysm sac for dCTA were 193.8 [136.3–225] and 47.3 [38.6–53.4], respectively. The median BF values in the endoleak and aneurysm sac for dCTP were 195.1 [136.5–214.6] and 11.2 [8.1–12.8] mL/100 mL/min, respectively. The median endoleak indexes derived from dCTP and dCTA were 18.5 [10.8–20.5] vs 3.5 [5–2.7], respectively. The endoleak index provided by dCTP showed to be significantly higher compared to the one provided by dCTA (p = 0.02).

### Diagnostic confidence and endoleak index for dynamic CTA and CTP

The median values of diagnostic confidence provided by dCTA and dCTP for patients without endoleaks were 1.5 [1.5–1.5] and 1 [1–1], respectively; moreover, the confidence in ruling out the presence of an endoleak by dCTP was significantly superior (p < 0.01) compared to that provided by dCTA. With respect to positive patients, the median values of diagnostic confidence for dCTA and dCTP were 4.5 (4–5) and 5 (5–5), respectively. Similarly, dCTP showed a better diagnostic confidence in the detection of endoleaks in comparison with dCTA, even though this difference was not statistically significant (p = 0.11).

### Inter- and intra-observer agreement for dynamic CTA and CTP

The inter-observer agreement for dCTP and dCTA was excellent: κ = 0.93 [0.82–1.0] for the first read and κ = 1.0 [1.0–1.0] for the second read. Similarly, the intra-observer agreement for dCTP and dCTA was also very good: κ = 1.0 [1.0–1.0] for observe A and κ = 0.93 [0.82–1.0] for observer B.

### Analysis time

The mean time to formulate the diagnosis with dCTA was 242 ± 51.2 seconds and such was significantly lower (p < 0.01) than the time spent to reconstruct and analyze the dCTP images (262 ± 45.6 seconds). Although, considering 188.6 ± 16.8 seconds were necessary to reconstruct perfusion maps from the dCTP dataset, a mean time of 73.4 ± 55.6 seconds was spent diagnosing with this technique.

### DLP and effective radiation dose

The median DLP and effective radiation dose values of the tri-phasic acquisitions and dCTA are reported in Table 4. The effective radiation dose of tri-phasic CTA was significantly lower compared to the dynamic one (12.4 [8.9–14.9] vs 15.9 [15.9–16] mSv, respectively; p = 0.01).

**Table 4:** Dose length product and effective radiation dose of tri-phasic and dynamic CTA scans.

	Non-contrast phase	Arterial phase	Delayed phase	Complete tri-phasic scan	Dynamic CTA
Dose length product (mGy/cm)	365.5 [279.8–448.8]	241.2 [204.8–288]	241 [204.5–288.2]	829.0 [596.8–994.3]	1063.8 [1063.6–1064.9]
Effective radiation dose (mSv)	5.5 [4.2–6.7]	3.6 [3.1–4.3]	3.6 [3.1–4.3]	12.4 [8.9–14.9]	15.9 [15.9–16]

Values are expressed with median and [interquartile ranges].

## Discussion

The purpose of this investigation was to assess the feasibility of a quantitative analysis of dCTA for the detection of endoleaks. In this study, we showed that dCTA enabled complete agreement for endoleak detection on a per-patient basis compared to standard CTA.

**Fig 4:**
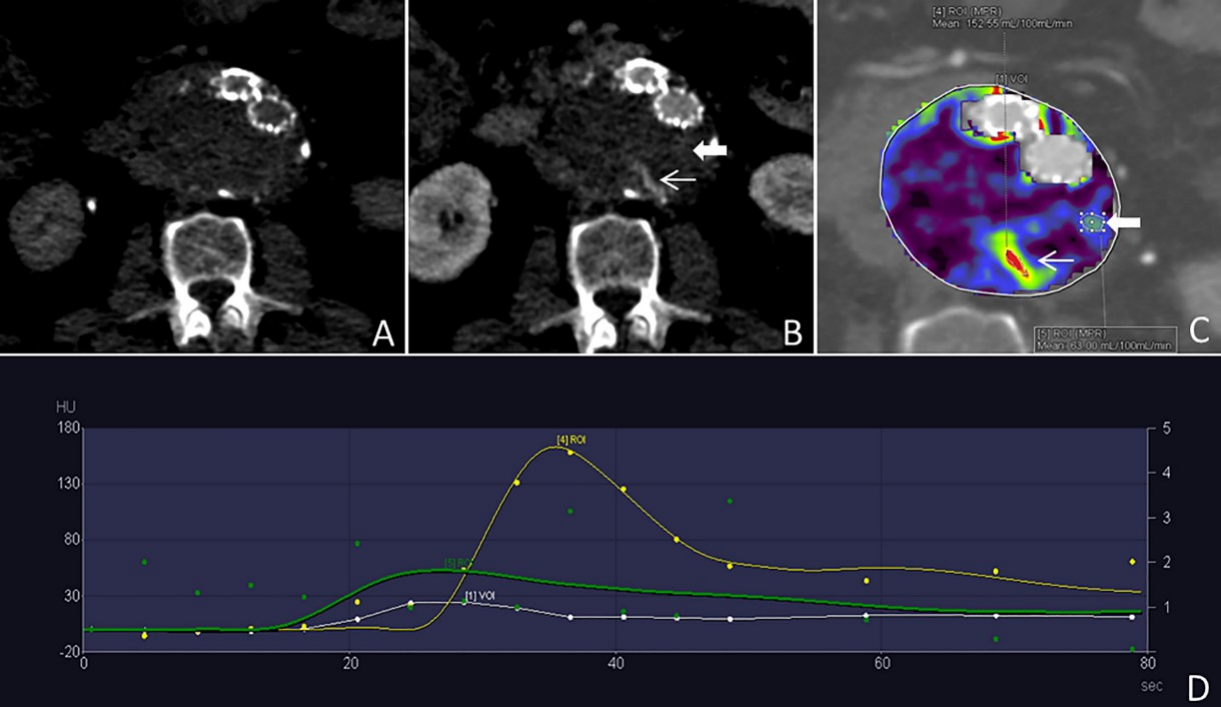
Discrimination between true and false endoleaks. This figure shows the same patient as in Fig 2. dCTA images acquired at 2 and 38 seconds after the contrast media injection (A and B, respectively) show a type II endoleak and a suspected inhomogeneous area in the aneurysm sac (thick and thin arrow, respectively in B). The color-coded perfusion map shows the true endoleak and the suspected lesion correspondent to the inhomogeneity in Panel B (thick and thin arrow, respectively in C); the TACs (D) allow to discriminate between the true (ROI 4) and the false endoleak (ROI 5): they demonstrate the typical kinetics for the former while the latter lacks the wash-in, the peak, and the wash out phase, the dots of the correspondent curve being randomly spread on the graph.

The dCTP approach provided high diagnostic confidence in detecting or excluding the presence of endoleaks (5 and 1, respectively). Notably, dCTA has proven to provide high diagnostic confidence in endoleak assessment, but we showed that dCTP can yield a significantly higher confidence for endoleak rule-out (p < 0.01) [18]. On the contrary, diagnostic confidence from dCTP was not significantly greater (p = 0.11) with respect to endoleak detection. We assume that this improvement in diagnostic confidence may be due to the qualitative and quantitative assessment enabled by dCTP through color-coded maps based on absolute BF values or the TAC correspondent to a suspected lesion. Therefore, this method allows for easier discrimination between true and false endoleaks (Fig 4). Additionally, we calculated the ratio between the BF and HU values of endoleaks and the aneurysm sac to assess the capability and reliability of the two techniques to discriminate endoleaks from the surrounding tissue. The evaluation of BF yielded a significantly higher endoleak index in comparison to the HU values of the dCTA (18.5 [10.8–20.5] vs 3.5 [5–2.7], p = 0.02).

On a per-lesion basis, dCTP enabled identification of an extra endoleak in one patient, with confirmation by visual assessment of the dCTA images. This finding is in accordance with a previous study that showed that dCTA has a higher endoleak detection rate compared to the static acquisition [14]. In another patient, the observers interpreted a single endoleak fed by the inferior mesenteric and a lumbar artery as two endoleaks; yet, the dynamic and triphasic CTA classified it as a single lesion. We believe that this misinterpretation resulted from the complex anatomy and filling kinetics of this endoleak; in effect, the presence of two feeding vessels yielded two time-peaks at the cranial and caudal portion of this endoleak (36 and 48 seconds, respectively), suggesting two different lesions. With respect to the endoleak that was not identified by the triphasic CTA, we believe that the limited temporal window of the static acquisitions hindered its detection with the arterial and venous phase (7 and 53 seconds after the bolus tracking threshold, respectively) [19,20]. In fact, this endoleak was characterized by a time peak of 36 seconds and a rapid washout.

Regarding the efficiency associated with dCTP, the mean time spent analyzing the perfusion maps was 73.4 ± 55.6 seconds. Considering the automatic nature of this reconstruction process (188.6 ± 16.8 seconds), the total time spent during the entire process (242 ± 51.2 seconds) could shorten considerably with higher computer power.

We also found that the radiation dose in our dCTA protocol is significantly higher than that of the triphasic scan (15.9 vs 12.4 mSv, p = 0.01). However, we are confident that limiting the z-axis coverage to contain only the endoprosthesis and aneurysm sac and specifically tailoring each protocol to patient-specific tube current and kV modulation can result in a substantial dose reduction. On the other hand, this investigation showed that our dCTA protocol promotes high values of CNR for dCTA (24.7 ± 12 and 5 ± 3.3 for the aortic lumen and endoleak, respectively) and high subjective overall image quality values for dCTP while administering a reduced dose of contrast material using a low tube voltage.

The clinical relevance of monitoring endoleaks, especially type II endoleaks, after EVAR has not been demonstrated and is poorly understood. While type I and III endoleaks need definitive treatment, the management plan for type II endoleaks depends on e.g. the growth rate of the aneurysm sack. Such complications may potentially be identified by ultrasound, however, advanced imaging techniques, such as triphasic CT may be used for better visualization and treatment planning. The dynamic CT technique presented here provides quantitative measures that may improve our ability to identify and monitor otherwise undetected endoleaks, and provide more insights into endoleak management. However, the clinical relevance of these new dynamic and quantitative methods need to be evaluated in a wider range of prospective studies in order to assess their potential e.g. to predict aneurysm sack growth in type II endoleaks. In addition, such future studies should investigate if quantitative perfusion measures in endoleaks can be used to generate cut-off values that may help to determined which endoleaks need treatment. Finally, the ability of dCTA and dCTP to identify new or additional endoleaks and their potential clinical relevance are important to be investigated in studies using either time-resolved MRA or invasive angiography as reference standard. It also needs to be mentioned, however, that the general availability of dynamic CT is limited and mostly depends on hardware. In clinical routine, dynamic acquisition is widely used for the imaging of stroke patients, however, imaging larger field of view (larger than the detector coverage) requires dedicated techniques, such as shuttle mode acquisition that we used in our study. Shuttle mode is able to increase detector coverage by moving the table back and forth during acquisition. The availability of shuttle mode is mostly limited to dual source systems.

This investigation has several limitations that warrant consideration. Our single-center study included a limited number of patients; future multicenter prospective studies should describe the larger scale utility and prognostic value of applying dCTP in this clinical scenario. The study patients only had type II endoleaks; hence, we were unable to investigate the value of dCTP in the evaluation of the other types of endoleaks, particularly type I and III that, whenever present, require prompt treatment [21]. Our study did not have a reference standard, considering that the currently used standard of care CTA has limitations in enodleak detection and invasive angiography studies were not available in this patient population. Another potential limitation is the calculation method for blood flow inside the aneurysm sac. This computational method included a deconvolution and upslope calculation based on the CT Myocardial Perfusion software. This method, together with a limited sampling rate, may cause an underestimation of BF [22,23]. Moreover, further research is needed to determine which tracer kinetic model (we used the two-compartment one) best approximates the true value of the endoleak BF in the thrombotic tissue.

In conclusion, quantitative dCTP imaging enables detection of endoleaks and demonstrates a higher detection rate in comparison to triphasic CTA, in strong correlation with the visual assessment of dCTA studies.

## Supporting information

S1 Data Database demographics 5.0 new. (XLSX)

S2 Data Paper database Endo1. (XLSX)
